# Glycerol‐3‐phosphate acyltransferase 3‐mediated lipid droplets accumulation confers chemoresistance of colorectal cancer

**DOI:** 10.1002/mco2.486

**Published:** 2024-02-09

**Authors:** Ying Wang, Caihua Xu, Xianfeng Yang, Xiaofei Liu, Zijian Guo, Xinyu Lin, Lihua Li, Zhaohui Huang

**Affiliations:** ^1^ Wuxi Cancer Institute Affiliated Hospital of Jiangnan University Wuxi Jiangsu China; ^2^ Department of Oncology The First Affiliated Hospital of Soochow University Suzhou Jiangsu China; ^3^ Department of Radiology The First Affiliated Hospital of Soochow University Suzhou Jiangsu China; ^4^ Department of thyroid breast surgery, First Clinical College Shandong University of Traditional Chinese Medicine Jinan Shandong China; ^5^ Department of Oncological Surgery Affiliated Hospital of Jiangnan University Wuxi Jiangsu China

## Abstract

Colorectal cancer (CRC) is the third most common malignancy worldwide. It is well known that lipid metabolism reprogramming contributes to the tumor progression. However, the lipid metabolic alterations and potential remodeling mechanism underlying the chemoresistance of CRC remain largely unclear. In this study, we compared the gene expression profiles of chemoresistant versus control CRC cells from the GEO database and identified a key factor, Glycerol‐3‐phosphate acyltransferase 3 (GPAT3), that promotes lipid droplet (LD) production and confers chemoresistance of CRC. With applying of HPLC–MS and molecular dynamics simulation, we also demonstrated that the activity of lysophosphatidic acid synthesis by GPAT3 was dependent on its acetylation at K316 site. In particular, GPAT3‐mediated LD accumulation inhibited immunogenic cell death of tumor, and thus facilitated CD8+ T‐cell exhaustion and malignant progression in mouse xenografts and hepatic‐metastasis tumors in CRC patients. High GPAT3 expression turned CRC cells into nonimmunogenic cells after (Oxaliplatin) Oxa treatment, which was supported by a decrease in cytotoxic IFN‐γ release and CD8+ T‐cell exhaustion. In conclusion, these findings revealed the role of GPAT3‐associated LD accumulation, which conferred a malignant phenotype (chemoresistance) and regulated the tumor microenvironment of CRC. These results suggest that GPAT3 is a potential target to enhance CRC chemosensitivity and develop novel therapeutic interventions.

## Graphical abstract

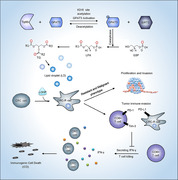

## 1 INTRODUCTION

Colorectal cancer (CRC) is the third most common malignancy. There are approximately 1880,725 new cases and 915,880 new deaths per year worldwide according to Global Cancer Statistics 2020.^1^ Metabolic reprogramming is a frequent event in tumor aggressiveness.^2^, ^3^, ^4^, ^5^ Except for Warburg effect, lipid remodeling in tumors mainly consists of de novo lipogenesis or cholesterogenesis due to oncogene‐initiated lipogenic enzyme overexpression, such as FASN and HMGCR.^3^ Excessively synthesized lipids in tumor mainly participate in membrane biogenesis, production of lipid droplets (LDs) and lipid‐derived second messengers. These biological processes (BPs) support tumor proliferation and malignant progression.^6^, ^7^, ^8^, ^9^

LDs are dynamic organelles that store energy and sustain lipid homeostasis. LD accumulation in nonadipocytic tissues is considered as a new hallmark of tumor.^7^ In CRC cells, LDs are crucial players in tumorigenicity and development.^10^ They were also reported to participate in resistance to drug therapy.^11^ Chemotherapeutics treatment significantly increased LD content.^12^ In 5‐FU or Oxaliplatin (Oxa)‐treated CRC cells, researchers observed marked LD accumulation and pointed out that excessive lipids protected tumor cells from oxidative stress damage by drugs.^13^ Indeed, LD accumulation was frequently found in drug‐resistanT‐cell lines.^14^, ^15^, ^16^, ^17^ Disclosing the potential mechanism underlying aberrant lipid metabolism triggered drug‐resistance is meaningful to develop novel therapeutic interventions and benefit CRC patients.

Immunogenic cell death (ICD) is a particular form of regulatory cell death (RCD) induced by antitumor agents or virus infection. It is triggered by stress. ICD could induce adaptive immunity against the antigen from dying cells. Dying tumor cells activate CRT exposure, immune cell recognition and CD8+ T‐cells killing effect.^18^ The dying signal transduction cross‐prime infiltrated CD8+ T lymphocytes to secrete cytotoxic cytokines such as IFN‐γ.^19^, ^20^ LDs were found playing an important role in this ICD‐related signal transduction.^13^ They might sequester CRT antigen and attenuate T‐cell killing effect on tumor cells.

In the present study, we screened out a key factor GPAT3 from expression profile of chemoresistant versus control CRC model using edgeR package. GPAT3, glycerol‐3‐phosphate acyltransferase 3, was an essential liposynthase in glycerol phosphate pathway. Its downstream metabolites, lysophosphatidic acid (LPA) and triacylglycerol (TG), were important materials for LD production. We identified a previously undiscovered link between GPAT3‐mediated LD accumulation and tumor resistance to chemotherapy in CRC, with applying HPLC–MS, molecular dynamics simulation, lipidomics, immunoprecipitation, immunofluorescence, flow cytometry, and so on. We proved that GPAT3 overexpression and excessive LD production facilitated CD8+ T‐cells exhaustion, immune evasion, and thus the malignant progression of CRC.

## 2 RESULTS

### 2.1 Differential expression and functional enrichment analyses for chemoresistance signature in CRC cells

**FIGURE 1:**
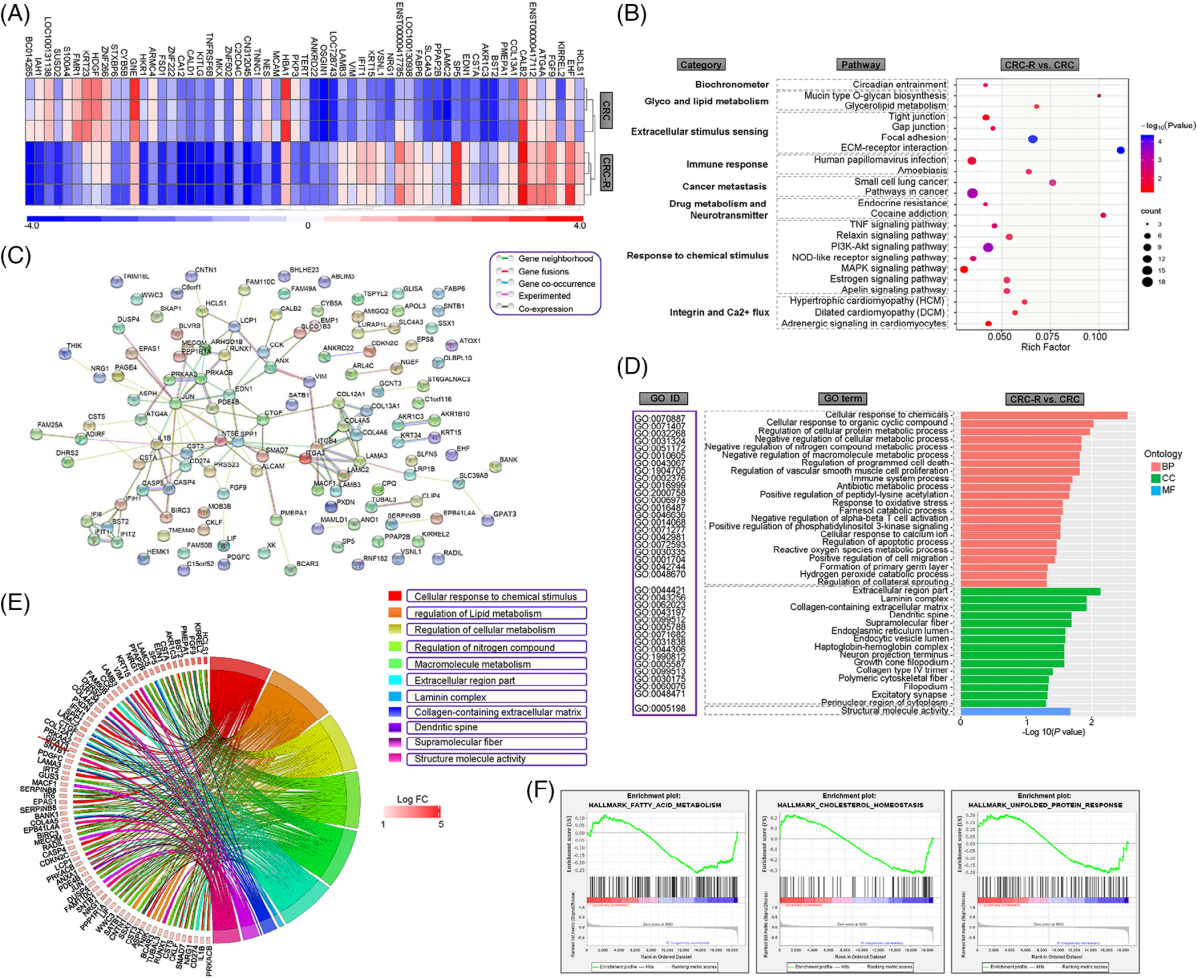
DEG, GO, pathway, PPI network, and GSEA enrichment analyses for chemoresistant versus control CRC cells from GEO datasets. (A) The hierarchical cluster heatmap of DEGs differentiates chemoresistant versus control CRC cells. Up and downregulated genes are marked by red and blue, respectively. (B) KEGG pathway analysis of DEGs. The *y*‐axis refers to pathway term. The *x*‐axis is rich factor. (C) PPI network analysis of DEGs, which is conducted by STRING online tools. (D) GO analysis of DEGs. The *y*‐axis is the GO category. The *x*‐axis is −log_10_ (*p* value). (E) Circle plot depicting the mapping relationship between important signal pathways and hub genes in specific chemoresistance profiles. (F) The principal pathways of GSEA enrichment analyses.

The mRNA microarray profile of chemoresistant versus control CRC cells (GSE42387) was screened from GEO database (https://www.ncbi.nlm.nih.gov/gds/). CRC chemoresistant (CRC‐R) cells were derived from CRC cells (HCT‐116) with continuous exposure to gradually increasing Oxa concentrations for 241 days. The concentration range was 0.01–20 μM.^21^ It was the subcell line acquiring Oxa resistance. There were three duplicates for each group in this profile. To assess the differences between CRC‐R and CRC cells, the gene expression profiles were compared and 247 differentially expressed genes (DEGs) (FC > 2, *p* < 0.05) were chosen out. The hierarchical cluster heatmap of the mRNA microarray revealed that CRC‐R cells had a notably different gene expression signature from CRC cells. It was a chemoresistance signature. Overall, 152 genes including HCLS1, EHF, PMEPA1, PPAP2B, FABP6, and GPAT3 showed obvious upregulation; 95 genes (such as IAH1, S100A4, HDGF, CYB5B, GNE, and KITLG) exhibited significant downregulation in CRC‐R cells (Figure 1A). We further performed gene functional studies for these DEGs of the chemoresistance signature. In the Kyoto Encyclopedia of Genes and Genomes (KEGG) pathway analysis, the pathways were significantly enriched in circadian entrainment (path:04713), mucin type O‐glycan biosynthesis (path:00512), glycerolipid metabolism (path:00564), and tight junction (path:04530) (Figure 1B). For Gene Ontology (GO) analysis, DEGs were strikingly enriched in three categories: (1) BP: cellular response to chemicals (GO:0070887), cellular response to organic cyclic compound (GO:0071407), regulation of cellular protein metabolic process (GO:0032268), and negative regulation of cellular metabolic process (GO:0031324); (2) cellular component (CC): extracellular region part (GO:0044421), laminin complex (GO:0043256), collagen‐containing extracellular matrix (GO:0062023), and dendritic spine (GO:0043197); (3) molecular function (MF): structure molecule activity (GO:0005198). GPAT3 was associated with cellular response to chemical stimulus and macromolecule metabolism (Figures 1D and E). The protein–protein interaction (PPI) network results were shown in Figure 1C. Gene set enrichment analysis (GSEA) illustrated that the principal pathways of chemoresistance‐related DEGs were fatty acid metabolism, cholesterol homeostasis and unfolded protein response (Figure 1F).

### 2.2 Screening and functional analyses for the key gene GPAT3 in CRC chemoresistance

**FIGURE 2:**
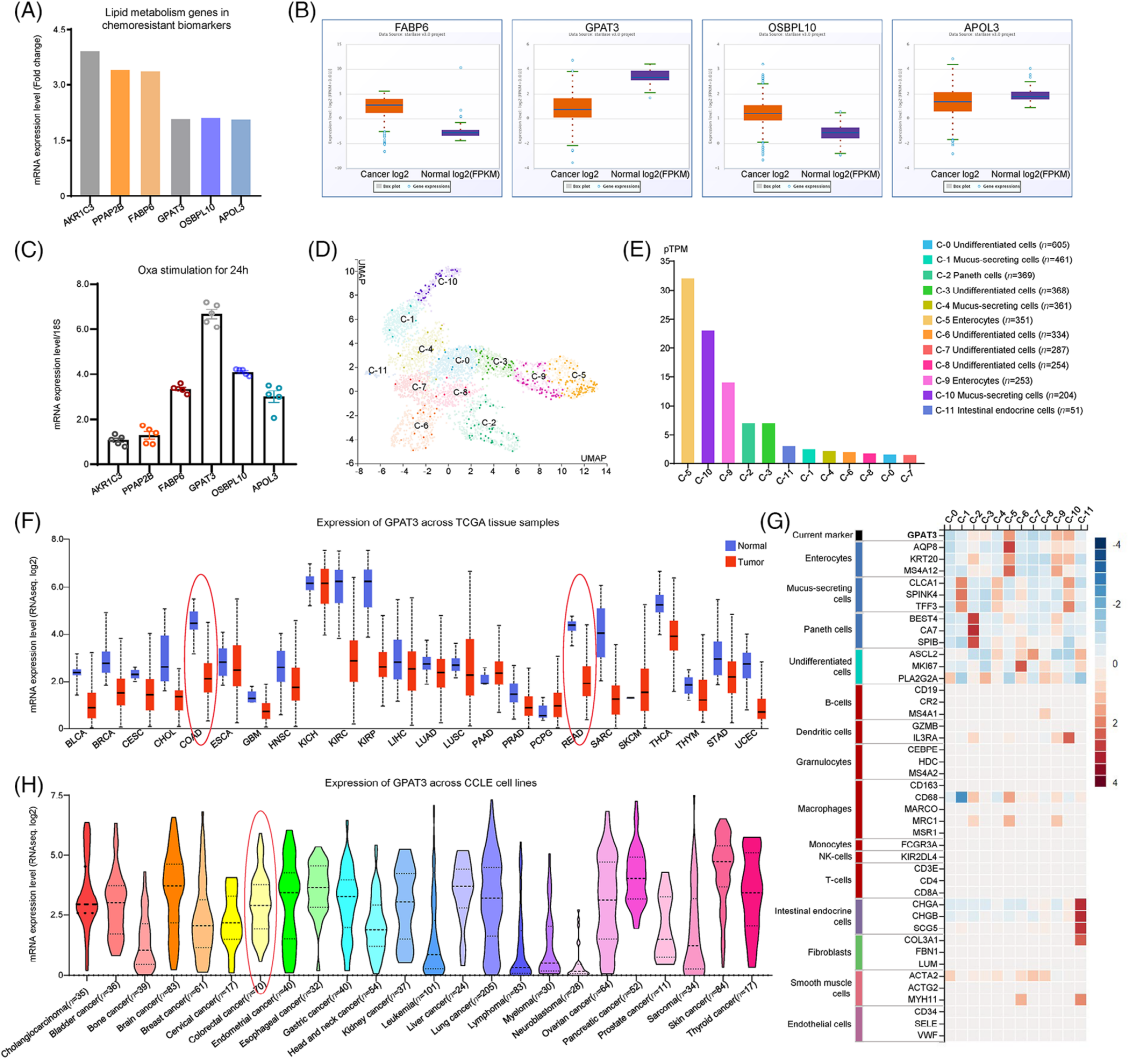
GPAT3 screening from lipid metabolism genes in chemoresistant biomarkers and GPAT3 expression characteristics. (A) Gene expression of lipid metabolism genes in chemoresistant biomarkers based on DEGs analysis. (B) FABP6, GPAT3, OSBPL10, and APOL3 expression in 471 colon adenocarcinoma (COAD) and 41 normal samples from TCGA database. (C) Relative mRNA expression of the six lipid metabolism genes in CRC cells after Oxa stimulation for 24 h. (D) The gene specificity and distribution of GPAT3 in each cell was clustered using cell RNA sequencing (scRNAseq) analysis of Atlas database. (The Single Cell Type section contains scRNAseq data from 25 major healthy tissues and organs and 444 individual cell type clusters.^34^) It was showed as interactive UMAP plot. (E) pTPM bar chart for GPAT3 expression in different‐cell type. (G) Heatmap for expression of GPAT3 and other cell markers in different‐cell type, such as mucus‐secreting cells, enterocytes. The low to high expression level was indicated from blue to red. (F) Expression of GPAT3 across TCGA tissue samples. (H) Expression of GPAT3 in cancer cell lines, using CCLE database.

Combining KEGG pathway analysis with GSEA, we observed that lipid metabolism, including glycerolipid metabolism, fatty acid metabolism, and cholesterol homeostasis, contributed most strikingly to the resistant phenotype of CRC. Thus, we focused on lipid metabolism‐related genes among the DEGs in the chemoresistance signature. Six genes containing AKR1C3, PPAP2B, FABP6, GPAT3, OSBPL10, and APOL3 were selected out. These genes were all overexpressed in CRC‐R cells (Figure 2A). Among these genes, GPAT3 and APOL3 were expressed at low levels in CRC, but they were obviously enhanced after Oxa stimulation for 24 h. The GPAT3 response to chemotherapeutic drug was the most intense (Figures 2B and C). Therefore, GPAT3 was considered as a key factor of chemoresistance and was included in further investigations. In expression analyses of TCGA tissue samples, GPAT3 mRNA levels were notably lower in colon and rectal carcinoma samples than in normal samples. Moreover, we analyzed GPAT3 expression according to RNA‐seq data from cell lines recorded in CCLE database. GPAT3 expression in CRC cells was lower than that in brain cancer, esophageal cancer, gastric cancer, and liver cancer cells (RNAseq.log2 = 2.93) (Figures 2F and H). Using the single‐cell expression data from SCEA (https://www.ebi.ac.uk/gxa/sc/home) and HPA (https://www.proteinatlas.org/) database, we perform the single cell transcriptome analysis of GPAT3. A total of 3898 cells from colorectum tissues were characterized into 12 cell clusters (c‐0–c‐11) based on different GPAT3 expression (Figures 1D and G). GPAT3 expression was significantly higher in enterocytes and mucus‐secreting cells (pTPM > 10), which discriminate them from Paneth cells, undifferentiated cells, and intestinal endocrine cells (pTPM < 10). According to the UMAP dimension reduction graph, Cluster 1, 4, and 10 cells were classified as mucus‐secreting cells. Cluster 0, 3, 6, 7, and 8 cells were categorized to undifferentiated cells. Cluster 5 and 9 were sorted out to be enterocytes. Cluster 2 were determined as Paneth cells (Figure 1D). Expression pattern of GPAT3 and other cell markers in the 3898 cells were listed in Figure 1G. These findings highlighted the significance of GPAT3 expression in colorectum tissues. GPAT3 could clearly distinguish cells with different functions, suggesting that GPAT3 was very pivotal in regulation for BP of colorectal cells.

### 2.3 GPAT3 supported LD accumulation and conferred chemoresistance of CRC

**FIGURE 3:**
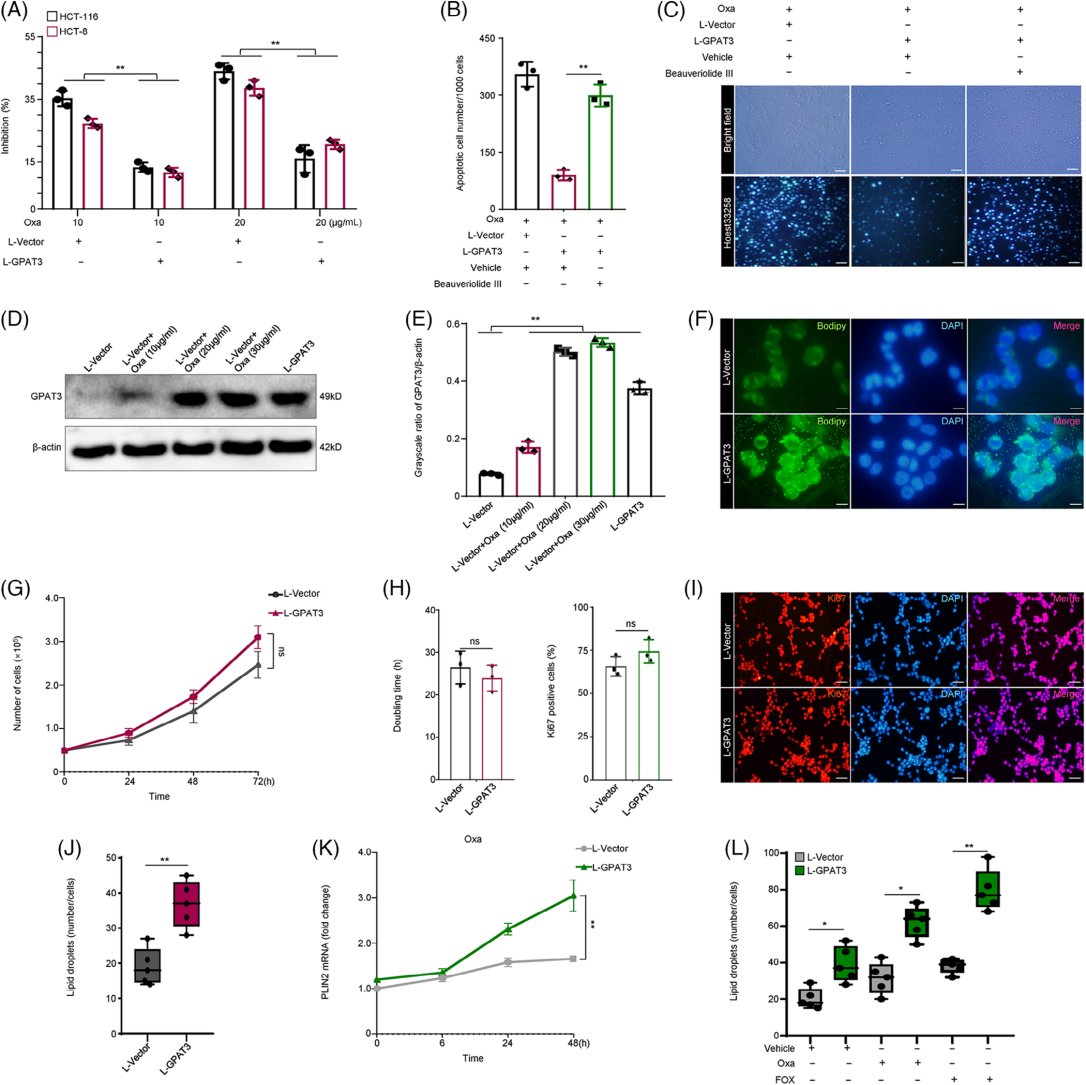
GPAT3‐mediated LD accumulation confers chemoresistance of CRC. (A) The growth inhibition impact of chemotherapeutic agent‐Oxa on CRC cells transducted with L‐GPAT3 or L‐Vector virus (for each group, *n* = 3). (B) Quantitative analysis of apoptotic cell in different groups (*n* = 3). (C) Representative graphs of apoptotic cells under different treatments in bright and fluorescence field. Scale bar = 100 μm. (D) GPAT3 expression in L‐GPAT3 cells and control cells treated with Oxa (10–30 μg/mL). (E) Densitometric analysis for (D). (F) L‐GPAT3 and L‐Vector cells were stained by Bodipy 493/503, a LD labeling probe (*n* = 3). Scale bar = 50 μm. (G) Growth curves of L‐GPAT3 versus L‐Vector cells (*n* = 3). (H) Left: Doubling times of L‐GPAT3 versus L‐Vector cells (*n* = 3). They were calculated according to the formula: DT = (*T* − *T*_0_) × (log2)/(log*N* − log*N*_0_). Right: Quantitative analysis for (I). (I) Representative images of Ki67 staining for L‐GPAT3 or L‐Vector groups (*n* = 3). Scale bar = 100 μm. (J) LD content analysis for (F). (K) Relative mRNA expression of PLIN2 at 0, 6, 24, and 48 h after Oxa treatments (*n* = 3). (L) LD content at 48 h of Oxa/FOX treatments in L‐GPAT3 versus L‐Vector cells (*n* = 3). *p < 0.05, **p < 0.01; ns, not significant. Error bars denoted s.e.m.

To further elucidate the role of key factor GPAT3 screened from CRC chemoresistance signature, we detected its effects on cell chemosensitivity and chemotherapy agent‐induced cell death. First, L‐GPAT3 and L‐Vector virus transducted CRC cells were examined using CCK‐8 assay for their sensitivity to Oxa. The IC50 of L‐GPAT3 virus transducted HCT116 cells were 82.6 μg/mL, compared with 29.1 μg/mL of L‐Vector cells. Ectopic expression of GPAT3 obviously enhanced chemoresistance of CRC cells to Oxa (Figure 3A). Under treatment of Oxa at 10 μg/mL for 24 h, the number of apoptotic cells in L‐GPAT3 group was significantly lower than that in empty vector group (L‐GPAT3 vs. L‐Vector: 92 ± 12 cells/1000 cells vs. 352 ± 32cells/1000 cells) (Figures 3B and C). Next, we assessed the relationship between Oxa stimulation and GPAT3 expression. Accompanied by Oxa incubation from 10 to 30 μg/mL, GPAT3 expression was obviously elevated in control CRC cells (Figures 3D and E). GPAT3 was an essential lipid‐metabolic gene promoting LPA/TG synthesis. This lipid metabolism process provided raw materials for LD production. Thus, we further detected LD content in L‐GPAT3 and L‐Vector cells under Oxa treatment. Applying specific LD staining of Bodipy 493/503, we observed that ectopic expression of GPAT3 notably promoted LD production in CRC cells as compared with control cells (Figures 3F and J). In addition of Beauveriolide III, an inhibitor of LD biogenesis, the Oxa‐induced apoptotic cells in L‐GPAT3 group were dramatically increased than that in vehicle counterpart (L‐GPAT3+Beauveriolide III vs. L‐GPAT3+Vehicle: 323 ± 30 cells/1000 cells vs. 92 ± 12 cells/1000 cells) (Figures 3B and C). Altogether, these data suggested that the chemoresistance of CRC was dependent on GPAT3‐supported LD accumulation. Moreover, we detected the mRNA expression of perilipin 2 (PLIN2), a LD coat protein. It was a biomarker of rapid LD biogenesis.^22^ As shown in Figure 3K, with Oxa stimulation, GPAT3 significantly potentiated PLIN2 expression at 24 and 48 h in CRC cells. Accordantly, GPAT3 also accelerated the LD production in CRC cells with different chemotherapeutic treatments (Oxa and FOX) (Figure 3L). To reveal the potential connection between GPAT3 and CRC proliferation, we monitored cell proliferation using Ki67 staining and depicted the cell growth curve. To our surprise, despite the high‐LD content in L‐GPAT3 cells, they seemed not propagated more fast than low‐LD L‐Vector cells (L‐GPAT3 vs. L‐Vector: doubling times = 20.5 ± 1.54 vs. 26.4 ± 3.88 h, *p* = 0.07 > 0.05, ns; Ki67 positive cells = 74.3 ± 6.81 vs. 65.7 ± 5.69%, *p* = 0.17 > 0.05, ns) (Figures 3G–I).

### 2.4 GPAT3 was acetylated at K316 site by TIP60, and its acetylation promoted the LD production

**FIGURE 4:**
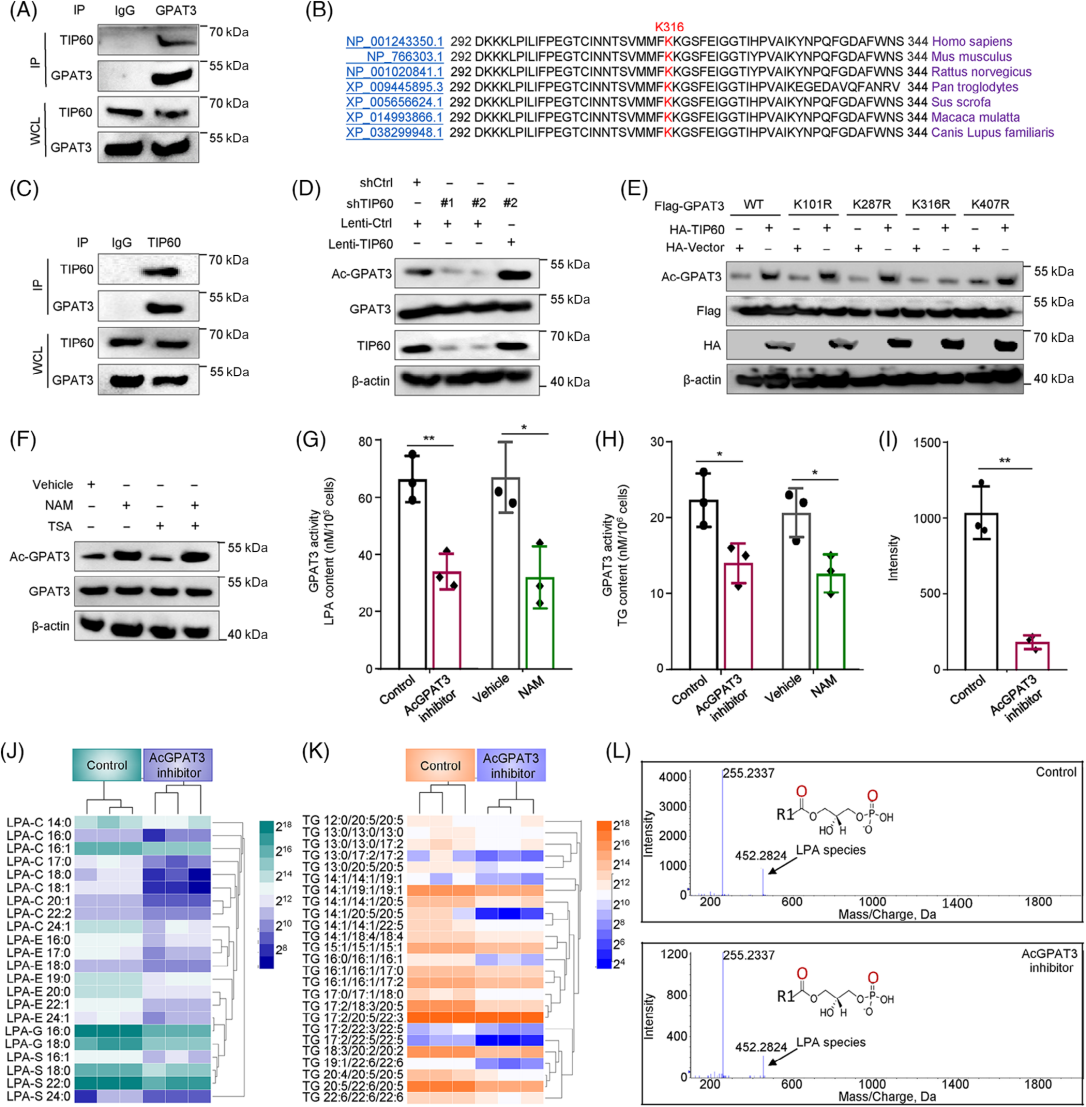
GPAT3 was acetylated at K316 site by TIP60, and its acetylation promoted the LD production. (A and C) Endogenous interaction of TIP60 and GPAT3. (B) Alignment of K316 and flanking amino acid sequence of GPAT3 between different species. (D) TIP60 inhibition reduced GPAT3 acetylation. (E) GPAT3 was acetylated by TIP60 at K316 site. (F) GPAT3 acetylation was enhanced by NAM instead of TSA. (G) GPAT3 activity detection by LPA quantification in different groups (*n* = 3). (H) GPAT3 activity detection by TG quantification (*n* = 3). Hierarchical clustering heatmap of LPA species (J) or TG species (K) concentration in lipidomic data from control and AcGPAT3 inhibitor groups. Up to downregulated genes are indicated using green‐to‐blue or orange‐to‐purple, respectively. (L) Verification of LPA concentration in Control and AcGPAT3 inhibitor groups with HPLC–MS and its quantification analyses (I) (*n* = 3). *p < 0.05, **p < 0.01. Error bars denoted s.e.m.

During the study of GPAT3 structure and function, we found that 4 putative acetylation sites of GPAT3 were predicted according to CSS‐Palm database and Protein Lysine Modifications Database (PLMD). For lipid metabolic process in different cells, acetylation of liposynthase was pivotal for its catalytic activity.^23^, ^24^, ^25^ Thus, we constructed Flag‐GPAT3 plasmids to further investigate the relationship between GPAT3 acetylation and its metabolic activity. First, we chose out the acetyltransferase of GPAT3 by performing coIP experiments. Among GCN5, TIP60, P300, and PCAF, TIP60 was confirmed to endogenously interact with GPAT3 in HCT‐116 cells (Figures S1A and 4A and C). We further observed that interfering TIP60 expression in CRC cells significantly reduced GPAT3 acetylation, but not GPAT3 protein level. However, ectopic expression of TIP60 exhibited the reverse effect (Figure 4D).

Based on the predicted acetylation sites of GPAT3 using online tools CSS‐Palm and PLMD, we generated four mutants from lysing (K) to arginine (R) at K101, K287, K316, and K407. As shown in Figure 4E, only K316R dramatically depressed TIP60‐triggered GPAT3 acetylation, suggesting that GPAT3 was acetylated at K316, while not K101, k287, and K407 (Figure 4E). In subsequent analysis, K316 was a high evolutionarily conserved site (Figure 4B). GPAT3 is a central enzyme in glycerol phosphate pathway. Its downstream metabolites, LPA and TG, are important lipids supporting LD production. Therefore, we detected the impact of GPAT3 acetylation on synthesis of downstream metabolites LPA and TG. Using ELISA kit, we found that the GPAT3 activity (showed as LPA content) in AcGPAT3 inhibitor group was markedly lower than that in control group (34.0 ± 6.24 nM/106 vs. 66.3 ± 8.08 nM/106 cells). As a verified effective reagent, NAM significantly inhibited GPAT3 deacetylation (Figure 4F), it thus inhibited GPAT3 activity and LPA/TG production (LPA: from 67.0 ± 12.29 nM/106 to 32.0 ± 10.82 nM/106 cells; TG: from 20.7 ± 3.21 nM/106 to 12.7 ± 2.52 nM/106 cells) (Figure 4G). Consistently, TG content in AcGPAT3 inhibitor group was obviously suppressed than that in control group (14.0 ± 2.65 nM/106 vs. 22.3 ± 3.51 nM/106 cells) (Figure 4H). Moreover, lipidomic analysis demonstrated that concentrations of LPA species (Figure 4J) and TG species (Figure 4K) were mostly decreased in AcGPAT3 inhibitor groups. We further verified the LPA concentration applying HPLC–MS and discovered that it reduced from 1035.0 ± 173.85 in control group to 182.7 ± 45.49 in AcGPAT3 inhibitor group (Figures 4L and I). These results suggested that inhibiting GPAT3 acetylation notably interrupted the metabolic activity of GPAT3.

### 2.5 GPAT3 was deacetylated at K316 site by SIRT3

**FIGURE 5:**
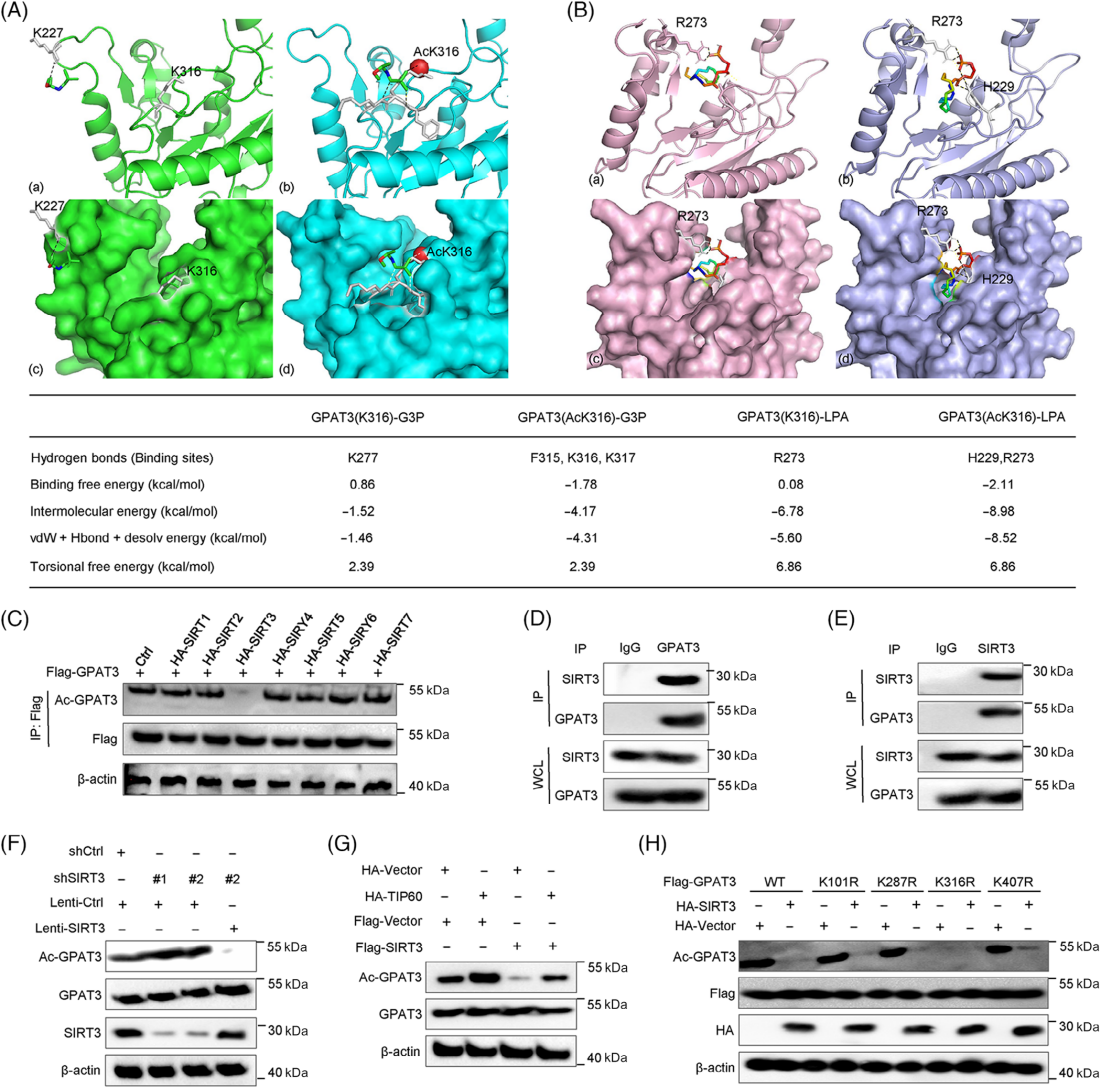
GPAT3 was deacetylated at K316 site by SIRT3. The different conformations of complexes for GPAT3 and substrate‐G3P (A) or metabolite‐LPA (B) in the unacetylated state (a, c) and acetylated state (b, d). The protein‐ligand binding complexes in ribbon (a, b) and surface (c, d) models. The molecular mechanics energies for the related residues in different conformations that presented favorable interactions with substrate‐G3P or metabolite‐LPA are listed in the table. (C) The coexpression of SIRT3 reduced GPAT3 acetylation, but not other SIRT molecules. (D and E) Endogenous interaction of SIRT3 and GPAT3. (F) Depletion of SIRT3 increased GPAT3 acetylation and overexpression of SIRT3 inhibited GPAT3 acetylation. (G) SIRT3 antagonizes Tip60‐promoted GPAT3 acetylation. (H) SIRT3 deacetylated GPAT3 at K316 site.

To understand the structural basis underlying K316 acetylation‐mediated variation of substrate affinity, we performed a molecular dynamics simulation using AutoDock and Amber biomolecular simulation. Two pair of molecular models of GPAT3–G3P and GPAT3–LPA were constructed based on the 3D X‐ray crystal structure. The left models (a and c) showed the unacetylated state with K316, and the right models (b and d) referred to the acetylated state with AcK316 (Figures 5A and B). To quantity the GPAT3 affinity to G3P and LPA in two different states, the molecular mechanics energies combined with the generalized born and surface area continuum solvation (MM‐GBSA)‐related binding free energy and the contribution of each surrounding residue were calculated. Binding free energies of GPAT3(K316)–G3P and GPAT3(AcK316)–G3P were 0.86 and −1.78 kcal/mol, respectively. Consistently, binding free energies of 0.08 and −2.11 kcal/mol were generated from GPAT3–LPA at K316 unacetylated and acetylated states, respectively (Figures 5A and B). This finding suggested that both G3P and LPA bound GPAT3 stronger in the acetylated state than that in the unacetylated state. It explained well that why GPAT3 metabolic activity was significantly higher at K316 acetylation state. As an orientation and conformation alteration with K316 acetylation, the dominant residues favoring G3P were transformed from K277 to F315, K316 and K317. Simultaneously, hydrogen bonds binding metabolite LPA were shifted from GPAT3 R273 to H229 and R273, presenting a better interaction. The conformation change was the main reason for higher substrate affinity of GPAT3 K316 acetylation. These results indicated that K316 acetylation regulated the direction of the GPAT3 reaction by altering substrate affinity.

We next examined the probable GPAT3 downstream deacetylase applying trichostatin A (TSA) and nicotinamide (NAM) to specifically inhibit HDAC and SIRT members, respectively. GPAT3 acetylation was obviously enhanced after incubation with NAM rather than TSA (Figure 4F). This finding indicated that a SIRT molecule likely contributed to GPAT3 deacetylation. Subsequently, we co‐expressed GPAT3 and SIRT1‐SIRT7 in SIRT family in HCT‐116 cells and observed that GPAT3 acetylation was dampened only by SIRT3 (Figure 5C). We further verified the endogenous interactions between GPAT3 and SIRT3 via coIP assays (Figures 5D and E). GPAT3 acetylation was enhanced by SIRT3 depletion, and repressed by SIRT3 overexpression (Figure 5F). To confirm the impact of TIP60 and SIRT3 on GPAT3 acetylation, we co‐expressed TIP60 and SIRT3 in CRC cells. As showed in Figure 5G, SIRT3 significantly diminished TIP60‐mediated GPAT3 acetylation. In particularly, the GPAT3 deacetylation by SIRT3 was blocked by K316R, but not other mutants (Figure 5H).

### 2.6 GPAT3‐induced LD accumulation promoted tumor progression and suppressed ICD

**FIGURE 6:**
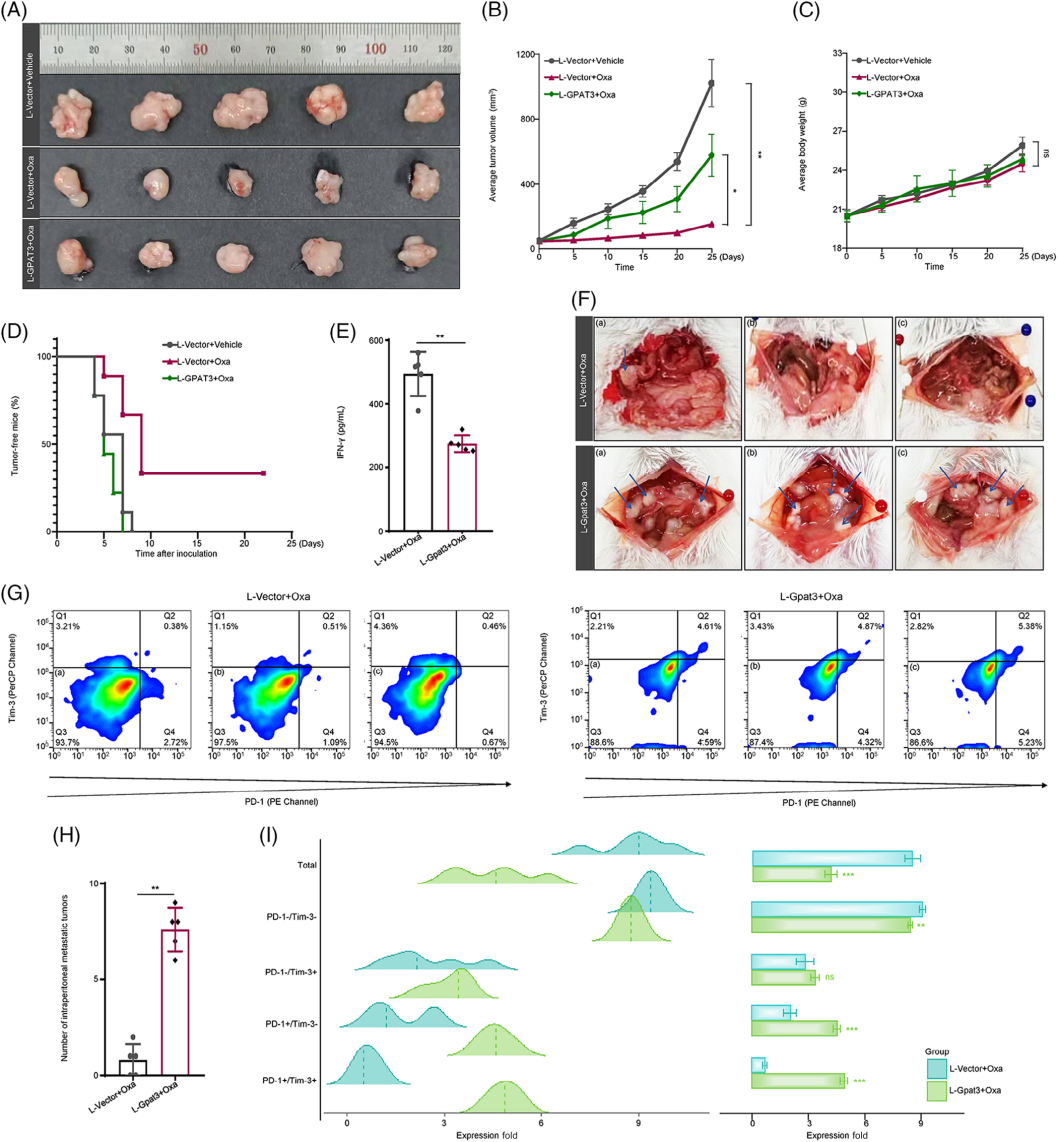
GPAT3‐induced LD accumulation promoted tumor progression and suppressed ICD. (A) Xenograft tumors of nude mice in L‐Vector + Vehicle, L‐Vector + Oxa, and L‐GPAT3 + Oxa groups (for each group, *n* = 5). (B) Average tumor volume and (C) body weight of xenograft mice models for each group. (D) Kaplan–Meier curves for tumor‐free mice in L‐Vector + Vehicle, L‐Vector + Oxa, and L‐GPAT3 + Oxa groups (*n* = 9 for each group). *p* Values were assessed by a log‐rank test. (E) The supernatant of fragmentized tumors from peritoneal metastasis models was assayed by ELISA for IFN‐γ production (*n* = 5). (F) Peritoneal metastasis tumors of L‐Vector and L‐Gpat3 mice (*n* = 5). (G) Representative scatterplots for flow cytometry analyses of PD‐1+Tim‐3+ cells in tumor infiltrated CD8+ T‐cells from peritoneal metastasis tumors in L‐Vector and L‐Gpat3 groups (*n* = 3). *p* Values were calculated using the multiple Student *t*‐test. (H) Quantitative analysis of (F). (I) Ridgeline plots and quantification analyses of (G). *p < 0.05, **p < 0.01; ns, not significant. Error bars denoted s.e.m.

To evaluate the effect of GPAT3‐induced LD accumulation on tumor aggressiveness under chemotherapy in vivo, we subcutaneously vaccinated the nude mice with L‐Vector or L‐GPAT3 cells, generating three groups: L‐Vector cells + Vehicle, L‐Vector cells + Oxa, and L‐GPAT3 cells + Oxa. The growth of xenografts in different groups was monitored every 5 days. The data revealed that chemotherapeutic drug significantly reduced tumor volume (L‐Vector + Oxa vs. L‐Vector + Vehicle: 153.0 ± 18.00 vs. 1021.3 ± 146.56 mm^3^). Nevertheless, L‐GPAT3 cells combined with Oxa treatment accelerated tumor proliferation by 377.1% (L‐GPAT3 + Oxa vs. L‐Vector + Oxa: 577.0 ± 130.43 vs. 153.0 ± 18.00 mm^3^) (Figures 6A and B). Of note, there was no significant alteration of body weights between different groups (Figure 6C). There results illustrated that GPAT3 conferred the chemoresistance of CRC cells to chemotherapeutic in vivo. In Kaplan–Meier analysis, there was no tumor‐free mice in L‐Vector + Vehicle and L‐GPAT3 + Oxa group at day 9, but there were approximately 33.3% tumor‐free mice in L‐Vector + Oxa group (Figure 6D). While we inoculated Balb/c mice by L‐Gpat3 transducted CT‐26 cells, the results were similar (Figures S2A and B). GPAT3 also increased tumorigenesis capacity of cells suffering chemotherapy. As the bioinformatical analyses suggested that GPAT3 played an essential role in modulating cell immunological function (Figures S1B–D), we further assessed the changes of tumor immune infiltration by abdominal metastases models. in growing Oxa‐treated tumors. L‐Gpat3 tumor bearing Balb/c mice presented less tumor infiltration of CD8+ T‐cell after drug injection compared with control mice (L‐Gpat3 + Oxa vs. L‐Vector + Oxa: 4.52 ± 1.23 vs. 8.84 ± 1.07%) (Figures 6G and I). This phenomenon prompted us to evaluate the activation or exhaustion status of CD8+ T‐cells. Dying tumor cells activate CRT exposure, immune cell recognition, and CD8+ T‐cells killing effect. Tumor infiltrated CD8+ T‐cells and CD8+ T‐cells activation were hallmark events of tumor ICD.^26^ While CD8+ T‐cells exhaustion indicated inhibited tumor ICD. We measured the membrane CRT signals and percentage of CD8+ T‐cells expressing programmed cell death‐1 (PD‐1) and T‐cell immunoglobulin and mucin‐domain containing‐3 (Tim‐3). PD‐1 and Tim‐3 were typical markers of T‐cell exhaustion.^27^ Upon Oxa induction, membrane CRT exposure was significantly attenuated in L‐Gpat3 tumors (Figure S2C); Tim‐3^+^PD‐1^+^ subpopulation was accumulated in L‐Gpat3 tumors than that in control group (L‐Gpat3 + Oxa vs. L‐Vector + Oxa: 4.86 ± 0.43 vs. 0.66 ± 0.29%) (Figures 6G and I), suggesting that chemotherapy‐activated T‐cell immune response was suppressed by GPAT3. Consistent phenomenon was observed by immunofluorescence (Figures S2F and G). It presented T‐cell exhaustion in tumors. This was reinforced by the phenomenon that Oxa could only stimulate IFN‐γ production in control group, but not L‐Gpat3 group (Figure 6E). However, T‐cell immune suppression was associated with tumor malignant phenotype, such as metastasis. We also evaluated the metastasis capacity of cells in L‐Gpat3 group by abdominal metastases models. It showed that Gpat3 (mouse) significantly promoted tumor metastasis under Oxa treatment, as compared with L‐Vector group (Figures 6F and H and S2D and E).

### 2.7 GPAT3‐conferred chemoresistance promoted tumor malignant progression and suppressed ICD in CRC‐HM samples

**FIGURE 7:**
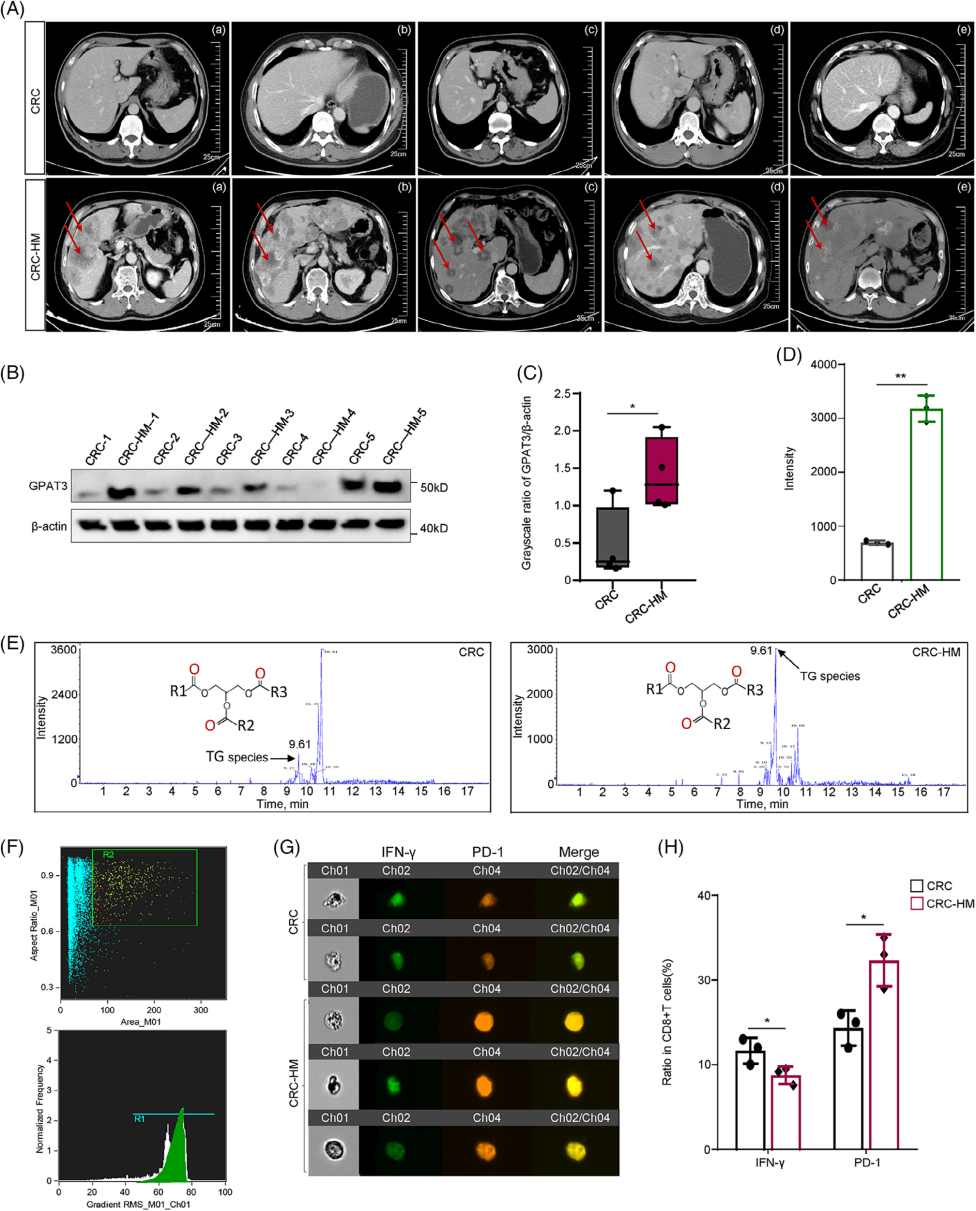
GPAT3‐conferred chemoresistance promoted tumor progression and suppressed ICD. (A) CT scanning images at the livers of patients in CRC or CRC‐HM groups (for each group, *n* = 5). (B) GPAT3 expression in specimens of patients from CRC or CRC‐HM groups (*n* = 5). (C) Quantitative analyses of (B). (E) TG concentration in CRC and CRC‐HM groups measured using HPLC–MS and its quantification analyses (D) (*n* = 3). (F) The SSC/FSC (side scatter/forward scatter) scatterplot and histogram of (G). (G) Cell images of tumor infiltrated CD8+ T in CRC and CRC‐HM groups scanned on Amnis® FlowSight Imaging flow cytometry platform (*n* = 3). (H) Quantifying analyses of (G). *p < 0.05, **p < 0.01; ns, not significant. Error bars denoted s.e.m.

To consolidate the relationship between GPAT3 overexpression/LD accumulation and chemoresistance/tumor immunosuppression, flow cytometry and HPLC–MS were performed on hepatic‐metastasis samples from CRC (CRC‐HM group) and control CRC (no recurrence/metastasis) patients. These patients were all received Oxa treatment and patients in CRC‐HM group were resistant to chemotherapy. Their computed tomography (CT) scanning images at livers were shown in Figure 7A. The GPAT3 expression were significantly higher in tissue samples from CRC‐HM group, as compared with that in CRC group (Figures 7B and C). Consistently, the LD component‐metabolite TG, was obviously accumulated in tissues of CRC‐HM group (Figures 7D and E). Flow cytometry analyses revealed that weak CD8+ T‐cell activity was presented in the CRC metastatic samples (Figures 7F–H). This suggesting that high GPAT3/LD production tumor exhibited an immune suppression microenvironment, which might facilitate tumor malignant progression.

## 3 DISCUSSION

In this study, we found that GPAT3 conferred CRC chemoresistance and inhibited tumor ICD through GPAT3‐supported LD accumulation. To our knowledge, this is the first study elucidating the role of GPAT3 in lipid metabolism and CRC chemoresistance.

GPAT3 is a central enzyme in the glycerol phosphate pathway. Its downstream metabolites LPA and TG, are important lipids supporting LD production. The mechanism by which GPAT3 conferred CRC chemoresistance might be interpreted into 3 aspects: First, GPAT3 overexpression might blunted the Oxa‐induced signal transduction. Second, GPAT3‐mediated LD production might be essential for the attenuation of drug‐induced tumor ICD and CD8+ T‐cell killing effects. Our study showed that GPAT3 expression significantly increased LPA/TG synthesis and thus LD accumulation. LD notably protected CRC cells from drug‐induced PD. GPAT3‐mediated cell protection of CRC from death could be abrogated by an LD inhibitor. Third, GPAT3 might trigger CD8+ T‐cell exhaustion in CRC, which was related to chemoresistance.^28^

ICD is one of RCD induced by antitumor agents or infection. It is triggered by stress. Dying tumor cells activate CRT exposure, immune cell recognition and CD8+ T lymphocytes killing.^18^ The dying signal transduction cross‐prime infiltrated CD8+ T lymphocytes to secrete cytotoxic cytokines such as IFN‐γ.^19^, ^20^ We demonstrated herein for the first time that high GPAT3 expression turned CRC cells into nonimmunogenic ones after Oxa treatment, which was supported by a decrease in cytotoxic IFN‐γ release and CD8+ T‐cells exhaustion. Tumor infiltrating CD8+ T‐cells and CD8+ T‐cells activation were hallmark events of tumor ICD.^26^ CD8+ T‐cells exhaustion indicated inhibited tumor ICD. Similar results were also found in other reports.^13^, ^29^ Lipid metabolic reprogramming regulated CD8+ T‐cell activity in tumor aggressiveness via inhibiting ICD.

We further explored the molecular mechanism of GPAT3 activation in lipid synthesis as a pivotal metabolic enzyme. Recently, numerous literatures suggested that the acetylation of liposynthase was significant for its catalytic activity.^23^, ^24^, ^25^ We demonstrated that GPAT3 was acetylated by TIP60 and deacetylated by SIRT3 using Co‐IP. Based on the predicted acetylation sites of GPAT3, we generated four mutants, and only K316R dramatically depressed TIP60‐triggered GPAT3 acetylation, Moreover, we verified the relationship between GPAT3 acetylation and metabolic activity. The results revealed that acetylated GPAT3 promoted the synthesis of its downstream metabolites LPA and TG. To understand the structural basis underlying K316 acetylation‐mediated variation of substrate affinity, we performed a molecular dynamics simulation. Two pairs of molecular models of GPAT3–G3P and GPAT3–LPA were constructed based on the 3D X‐ray crystal structure. It showed that GPAT3 affinity to G3P and LPA was significantly higher in acetylation state. The binding free energies of GPAT3(K316)–G3P and GPAT3(AcK316)–G3P were the lowest at 0.86 and −1.78 kcal/mol, respectively. It explained well that why GPAT3 metabolic activity was significantly higher at K316 acetylation state.

Cancer cells in CRC are heterogeneous. Postoperative chemotherapy might kill some CRC cells and screen cells with high GPAT3 expression out. These seed tumor cells were more aggressive than before. Targeting GPAT3‐mediated LD accumulation pathway might be helpful to develop novel therapeutic interventions to reverse chemoresistance of CRC patients.

**FIGURE 8:**
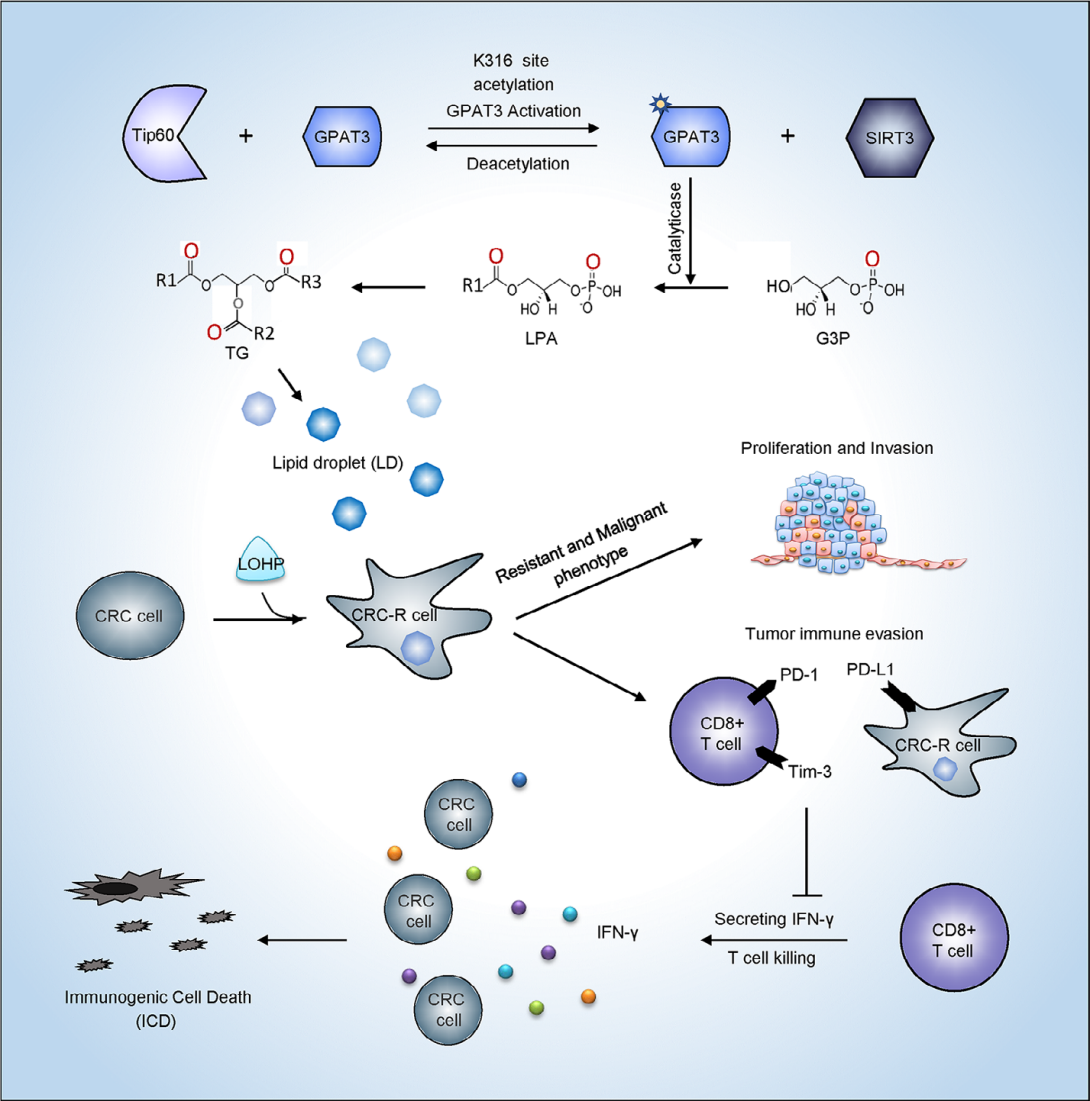
Schematic diagram for chemotherapy‐resistant CRC phenotype mediated by GPAT3 acetylation and lipid droplets (LDs) accumulation. GPAT3 is responsible for G3P converting to LPA. It is the first step in TG synthesis and LD production. Oxa induces GPAT3 overexpression and LDs accumulation, which confers chemoresistance to CRC cells. Chemoresistant cells present a more malignant phenotype and suppress the cell killing effect of CD8+ T‐cells, resulting in immune evasion and tumor recurrence.

In this investigation, we screened out a key factor GPAT3 from expression profiles of chemoresistant versus control CRC models. It was an essential liposynthase in catalyzing LD production. We identified a previously undiscovered link between GPAT3‐mediated LD accumulation and tumor resistance to Oxa in CRC. We showed both in vitro and in vivo that GPAT3 played an important role in LD accumulation, which conferred CRC chemoresistance by interfering tumor ICD. In conclusion, we proved that GPAT3 overexpression and excessive LD production facilitated chemoresistance, immune evasion, and thus the malignant progression of CRC (Figure 8).

However, there were limitations of the current study to consider: first, more advanced technologies should be applied to decode the microenvironment in chemoresistant CRC tissues, such as single‐cell sequencing and spatial transcriptome analyses. Second, CRC is a complex disease influenced by multiple genetic, environmental, and lifestyle factors. Animal models might not fully replicate the intricacies of human CRC. Differences in immune responses between mice and humans could potentially impact the clinical transformation of our research findings. Nowadays, organoids might be a novel choice. Thus, a dynamic, multidimensional research system for understanding the mechanisms between GPAT3‐mediated LD accumulation and CRC malignancy is still needed.

*Prospect*: Postoperative chemotherapy might kill some CRC cells and screen cells with high GPAT3 expression out. These seed tumor cells were resistant to chemotherapeutics and responsible for recurrence. Targeting GPAT3 mediated LD accumulation pathway might be a novel avenue to reverse chemoresistance and remodel tumor microenvironment.

## 4 MATERIALS AND METHODS

### 4.1 Data extraction

The transcriptome profiles and sample information of parental and chemotherapy‐resistant CRC cell lines were searched and downloaded from GEO database (https://www.ncbi.nlm.nih.gov/gds/). The RNA sequencing platform was Agilent‐014850 Whole Human Genome Microarray 4×44K G4112F (Agilent Technologies Inc., Santa Clara, CA, USA). CRC cells (HCT‐116, HT‐29, and LoVo) were cultured with gradually increasing drug (Oxa or SN‐38) concentration for ∼9 months, generating sub‐cell lines with acquired chemoresistance.

### 4.2 DEG, GO, and KEGG pathway analyses

DEGs from CRC‐R versus CRC model were selected out using R version 3.6.2 software (https://www.r‐project.org/) and edgeR package (http://www.bioconductor. org/packages/release/bioc/html/edgeR.html). DEGs were filtered according to FC≥2, *p* < 0.05. GO and KEGG enrichment were performed by STRING (https://string‐db.org/) and GEPIA (http://gepia.cancer‐pku.cn/) online tools. PPI network was analyzed with STRING.

### 4.3 Cell culture and GPAT3 transduction

Human CRC cell lines (HCT‐116, HCT‐8) and mouse CRC cell line (CT‐26) were purchased from American Tissue Culture Collection (ATCC) (Manassas, VA, USA) and cultured in DMEM supplemented with 10% FBS (Gibco, NY, USA), 100U/mL penicillin and 100 μg/mL streptomycin (Beyotime, Shanghai, China). GPAT3 lentiviral expression vector was constructed by inserting GPAT3 cDNA (Miaolingbio, Wuhan, China) into pCDH–CMV–MCS–EF1–copGFP vector (CD511B‐1; System Biosciences Inc., CA, USA) with *Eco*rRI and *Bam*HI sites. Forward primer: 5′‐GCGAATTCATGGAGGGCGCAGA‐3′; Reverse primer: 5′‐GCGGATCCTTAGCTGAGAGATCCA‐3′. pCDH–CMV–MCS–EF1–copGFP–GPAT3, lentiviral package plasmids pMD2.G and psPAX2 were transfected into HEK293 cells with a ratio of 4:2:3. The GPAT3 lentiviral (L‐GPAT3) particles in cell supernatant were purified and condensed using Universal Virus Concentration Kit according to the manufacturer's instructions (Beyotime). Empty vector and package plasmids derived lentiviral particles (L‐Vector) was used as control.

### 4.4 Chemoresistance evaluation

The L‐GPAT3 or L‐Vector cells were seeded in 96‐well plates (5 × 103 cells/well) and grew for 24 h. With treatment of Oxa (Yeasen biochem, Shanghai, China) at various concentration for 24 h, cell inhibition ratios were measured by cell counting kit‐8 assay (Dojindo Laboratories, Kumamoto, Japan) according to the manufacturer's instructions. The L‐GPAT3 or L‐Vector cells were treated with Oxa (10 μg/mL) for 24 h. The number of apoptotic cells/1000 cells were assessed applying Hoechst 33258 staining.^30^

### 4.5 Metabolites extraction

Cell pellets from AcGPAT3 inhibitor (MG149) and control groups were resuspended with 200 μL H_2_O+480 μL extract (MTBE:MeOH = 5:1). Then, cells were homogenized at 35 Hz for 4 min and sonicated on ice. This step was repeated for three times. The samples were incubated at −40°C for 1 h and centrifuged at 999 g for 15 min. Three hundred microliters of supernatant was transferred to a fresh tube and dried in a vacuum concentrator at 37°C. Next, the dried samples were reconstituted in 100 μL solution (methanol: dichloromethane = 1:1) by sonication on ice for 10 min. The samples were centrifuged at 18759 g for 15 min, and 75 μL supernatants were reserved for lipidomics analysis.

### 4.6 Lipidomics analysis

The HPLC separation was carried out using a ExionLC Infinity series UHPLC System (AB Sciex, USA), equipped with a Kinetex C18 column (2.1 × 100 mm, 1.7 μm; Phenomen). Mobile phase A: 40% H_2_O+60% acetonitrile+10 mmol/L ammonium formate. Mobile phase B: 10% acetonitrile+90% isopropanol + 50 mL 10 mmol/L ammonium formate/1000 mL mixed solvent. The HPLC analysis was carried with elution gradient as follows: 0–12.0 min, 40–100% B; 12.0–13.5 min, 100% B; 13.5–13.7 min, 100–40% B; 13.7–18.0 min, 40% B. The injection volume of samples was 2 μL.

The TripleTOF 5600 mass spectrometer was used for MS/MS spectra detection on an information‐dependent basis (IDA) with acquisition software (Analyst TF 1.7; AB Sciex). In each cycle, the most intensive 12 precursor ions (intensity > 100) were chosen for MS/MS at collision energy (CE) of 45 eV. Electron spray ionization source parameters were set as follows: gas 1 = 60 psi, gas 2 = 60 psi, curtain gas = 30 psi, declustering potential = 100 V, ion spray voltage floating (ISVF): 5000 V(pos)/−3800 V(Neg).

An in‐house program was performed using R for automatic data analysis of lipidomics. Raw data files were converted (wiff to mzXML format) using the “msconvert” program from ProteoWizard (version 3.0.19282). Then, the data were processed by LipidAnalyzer. Peak detection of MS1 data was conducted with the CentWave algorithm in XCMS. Lipid was identified through a spectral match with an in‐house MS/MS spectral library.

### 4.7 Molecular dynamic simulation

Based on X‐ray crystal structures of GPAT3, we generated complex models of acetylated/unacetylated GPAT–G3P and GPAT3–LPA by Amber biomolecular simulation package (http://ambermd.org/index.php). The partial charges of substrate, hydrogen bond, vdW, and torsional free energy were derived using antechamber module in Amber 20. Molecular dynamic simulations of GPAT3–G3P/LPA models were obtained in AMBER14SB force field. Energy was minimized by imposing a restraint on each GPAT3 and G3P/LPA complexes. Then, the whole system minimization was performed for a few thousand steps. NVT (particle number, volume, temperature) simulations: a 1 ns NVT dynamics was conducted and followed by a NPT (particle number, pressure, temperature) production run. During NPT, all hydrogen bonds were constrained by SHAKE algorithm. The cutoff value of nonbonded interactions and particle mesh was 10 A°. For each GPAT3 and G3P/LPA model, 3 independent molecular dynamic simulations were carried out with randomly generated velocities. Each trajectory was analyzed by cpptraj module in Amber 20.

### 4.8 Cell isolation and flow cytometry

Infiltrated CD8+ T‐cells were isolated from collagenase‐digested tumor tissues by a CD8+ T‐Cell Isolation Kit (#130‐096‐495, Miltenyi Biotec, Bergisch Gladbach, Germany) according to the instructions. Antibodies used in cell markers detection for CD8+ T‐cells activation/exhaustion: anti‐IFN‐γ‐FITC, anti‐Tim‐3‐PerCP, and anti‐PD‐1‐PE (eBioscience, San Diego, CA, USA). The experiments were performed as previously described.^31^

### 4.9 Immunoprecipitation and western blot

The open reading frame of human SIRT1‐7, TIP60, and GPAT3 (Miaolingbio) were subcloned into pcDNA3.1‐3×flag or pcDNA3.1‐HA vectors. Transfected cells were lysed with NP‐40 lysis buffer (Biosharp, Anhui, China). Anti‐AcGPAT3 were custom‐made from GL (GL Biochem, Shanghai, China) and applied for IP and Co‐IP assays. The immune precipitants eluted from SureBeads™ Protein A&G Magnetic Beads (Bio‐Rad Inc., Hercules, CA, USA) were examined by immunoblotting as described.^32^ Anti‐rabbit and mouse IgG (Beyotime) were used as negative controls. Anti‐TIP60, anti‐SIRT3, anti‐GPAT3, anti‐Flag, and anti‐HA (1:1000) were obtained from Abcam (Cambridge, MA, USA).

### 4.10 CRC tissue samples

A total of 20 CRC patients and 20 CRC‐hepatic metastasis (CRC‐HM) patient received Oxa treatment in The First Affiliated Hospital of Soochow University (2018–2021) were enrolled in this study. The clinical features of them were listed in Table S1. Patients in CRC‐HM group were resistance to chemotherapy. The diagnoses were pathologically and CT confirmed. Patients received preoperative chemotherapy and radiotherapy were excluded. The experiments were approved by the ethics committee of The First Affiliated Hospital of Soochow University. Each included patient signed an informed consent.

### 4.11 Xenograft models

A total of 30 eight‐week‐old male nude mice were divided into three groups: L‐Vector + Vehicle, L‐Vector + Oxa, L‐GPAT3 + Oxa (each group: *n* = 10). 1 × 107 L‐Vector or L‐GPAT3 virus stably transducted HCT‐116 cells were subcutaneously injected at flank region of nude mice. For experiments in Figure S2, 16 eight‐week‐old female Balb/c mice were divided into two groups: L‐Vector + Oxa, L‐Gpat3 + Oxa (each group: *n* = 8). 1 × 10^7^ L‐Vector or L‐Gpat3 virus stably transducted CT‐26 cells were subcutaneously injected at flank region of Balb/c mice. As the xenografts reached 50 mm^3^, Oxa (1.5 mg/kg) or vehicle were tail intravenous injected once every 2 days. The xenografts were measured by vernier caliper and the mice weights were monitored every 5 days. Twenty‐five days later, mice were sacrificed and tumor volume was calculated. All the protocols of animal experiments were approved by the Institutional Animal Care and Use Committee of Jiangnan University.

### 4.12 Peritoneal carcinomatosis models

A total of 20 six‐week‐old male BALB/c mice were intraperitoneally injected with L‐Vector or L‐Gpat3 (Gpat3: murine homologous gene of GPAT3) transducted CT‐26 cells; (5 × 10^6^cells/100 μL medium) (each group: *n* = 10). Oxa (1.5 mg/kg) were tail intravenous injected once every 2 days. The tumor‐vaccinated mice were sacrificed after 3 weeks. The peritoneal tumor nodes were calculated and compared.^33^ All the protocols of animal experiments were approved by the Institutional Animal Care and Use Committee of Jiangnan University.

### 4.13 Statistics

All statistical analyses were conducted by GraphPad 9.0 (GraphPad Software Inc., CA, USA). Data were represented as means ± s.e.m. and analyzed using Student's *t*‐test (two groups) or one‐way ANOVA (multi groups). Kaplan–Meier analyses with log‐rank test were performed to evaluate the tumor‐free survival. All statistical tests were two‐sided and *p* value < 0.05 was considered as significant.

## AUTHOR CONTRIBUTIONS

Ying Wang, Lihua Li, and Zhaohui Huang designed the study. Ying Wang, Caihua Xu, Xiaoqin Yang, and Xinyu Lin performed the bioinformatic analyses and experiments. Lihua Li provided the assistance for paper writing, revising, and submitting. Caihua Xu collected the clinical samples. Xianfeng Yang and Xiaofei Liu directed the clinical information analysis. Ying Wang and Zhaohui Huang wrote the manuscript and graphed the figures. All authors reviewed and proved the final manuscript.

## CONFLICT OF INTEREST STATEMENT

The authors have no conflict of interest about this work.

## ETHICS STATEMENT

All the protocols of animal experiments were approved by the Institutional Animal Care and Use Committee of Jiangnan University. The experiments using specimens and clinical data of CRC patients were approved by the ethics committee of The First Affiliated Hospital of Soochow University (No. 537).

## Supporting information

Supporting Information

## Data Availability

The authors declare that all the data supporting the findings of this study are available within the article and its supplementary information files or from the corresponding authors upon reasonable request.
