# Supplementary material for: Glycerol‐3‐phosphate acyltransferase 3‐mediated lipid droplets accumulation confers chemoresistance of colorectal cancer
Source: MedComm (2020). 2024 Feb 9;5(2):e486. doi: 10.1002/mco2.486 (PMC10857777; doi:10.1002/mco2.486)
Supplement: Supplementary file 1 — Supporting Information [file MCO2-5-e486-s001.docx]

**Glycerol-3-phosphate acyltransferase 3-mediated Lipid Droplets Accumulation Confers Chemoresistance of Colorectal Cancer**

Ying Wang^1#^*, Caihua Xu^2#^, Xianfeng Yang^3#,^ Xiaofei Liu^4^, Zijian Guo^5^, Xinyu Lin^1^, Lihua Li^1^*, Zhaohui Huang^1^*

^1^Wuxi Cancer Institute, Affiliated Hospital of Jiangnan University, Wuxi 214062, Jiangsu, China

^2^Department of Oncology, The First Affiliated Hospital of Soochow University, Suzhou 215006, Jiangsu, China

^3^Department of Radiology, The First Affiliated Hospital of Soochow University, Suzhou 215006, Jiangsu, China

^4^First Clinical College, Shandong University of Traditional Chinese Medicine, Jinan 250355, Shandong, China

^5^Department of Oncological Surgery, Affiliated Hospital of Jiangnan University, Wuxi 214062, Jiangsu, China

* Correspondence to: Zhaohui Huang, Wuxi Cancer Institute, Affiliated Hospital of Jiangnan University, Wuxi 214062, Jiangsu, China. Email: [zhaohuihuang@jiangnan.edu.cn](mailto:zhaohuihuang@jiangnan.edu.cn); Lihua Li, Wuxi Cancer Institute, Affiliated Hospital of Jiangnan University, Wuxi 214062, Jiangsu, China. Email: [llhwxsy@aliyun.com](mailto:llhwxsy@aliyun.com); Ying Wang, Wuxi Cancer Institute, Affiliated Hospital of Jiangnan University, Wuxi 214062, Jiangsu, China. Email: [wangying98620@jiangnan.edu.cn](mailto:wangying98620@jiangnan.edu.cn).

# These authors contributed equally to this work.

**Supplementary Figures:**

**
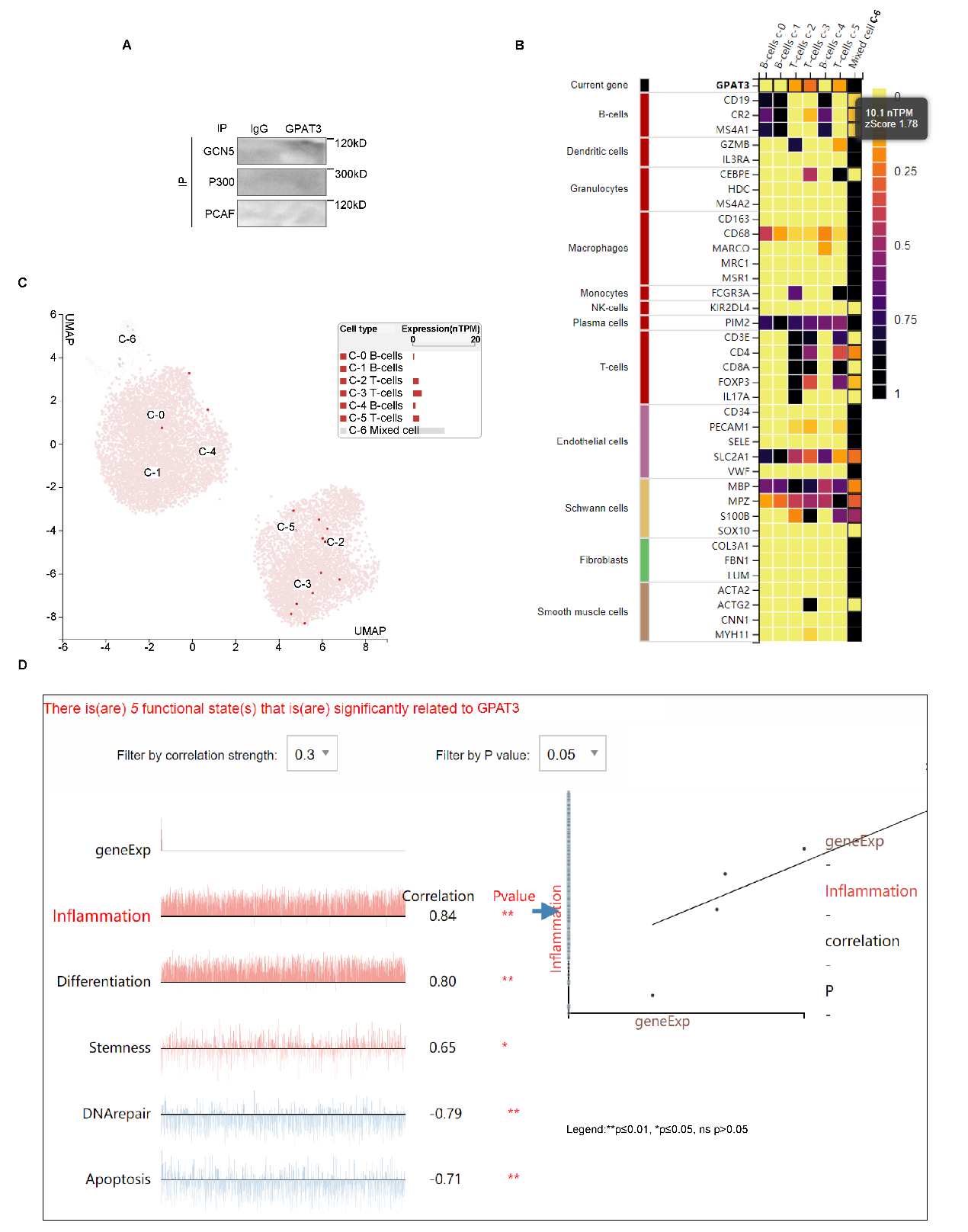
**

**Figure S1. Acetylase screening and bioinformatical analyses of GPAT3 on immunological function**

(A) CoIP detection for GPAT3 acetylase. (B) Heatmap for expression of GPAT3 and other cell markers in immune cells, such as B cells, macrophages, and T cells. The low to high expression level was indicated from yellow to black. (C) The interactive UMAP plot showing the gene specificity and distribution of GPAT3 in immune cells. The clustering was performed using cell RNA sequencing (scRNA seq) analysis of Atlas database. (D) GPAT3 was strongly correlated with inflammation function based on CancerSEA analysis.

**
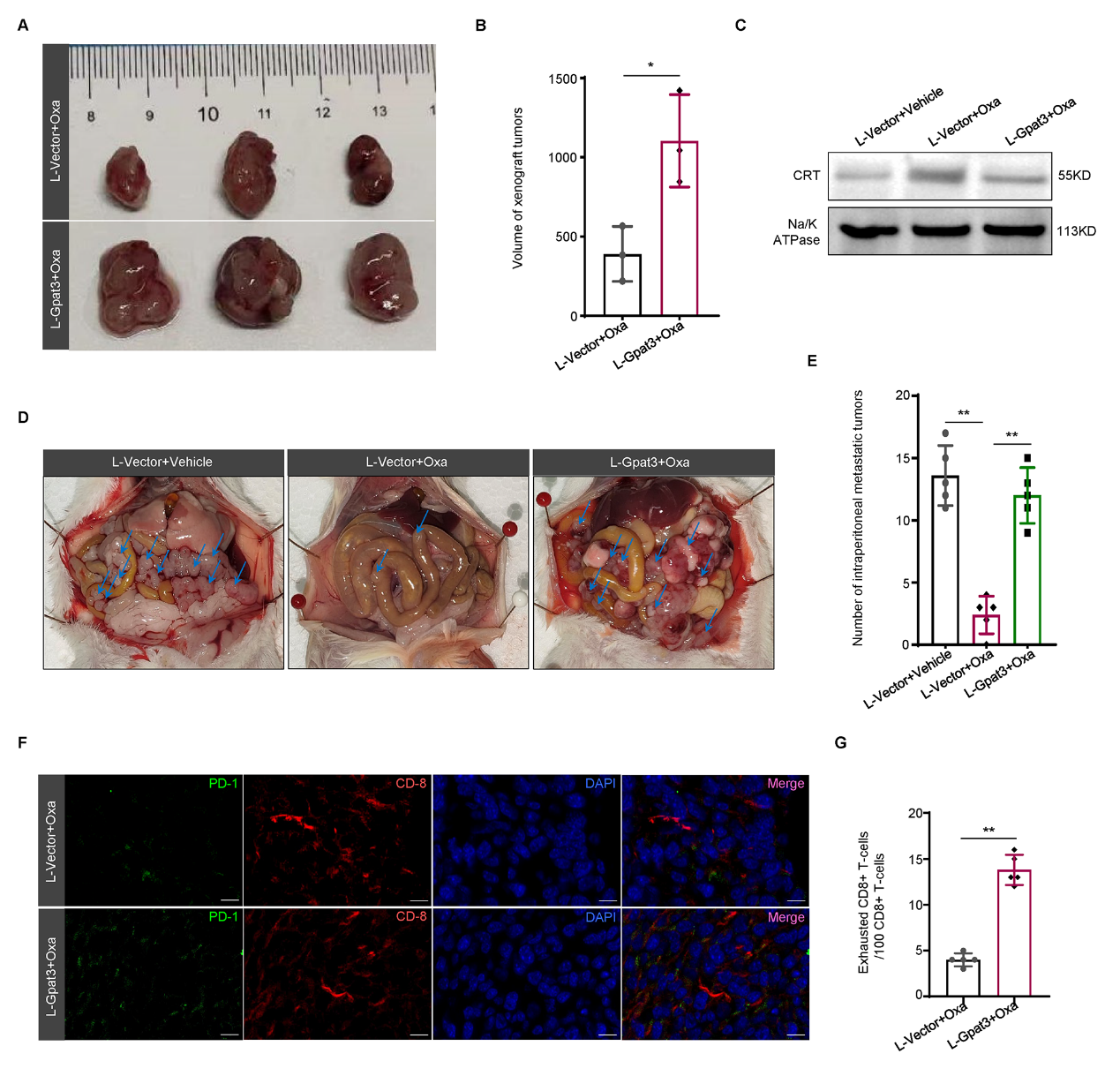
**

**Figure S2. GPAT3-mediated LD accumulation promoted tumor progression and suppressed ICD.** (A) Xenograft tumors of Balb/c mice in L-Vector+Oxa and L-GPAT3+Oxa cells (CT-26 cell). (B) 25 days later, average tumor volumes of xenograft mice models for each group. (C)Western blotting analysis for CRT expression in membrane protein of tumors from experiments (D). (D) Peritoneal metastasis tumors of L-Vector+Vehicle, L-Vector+Oxa and L-Gpat3+Oxa mice and quantitative analysis (E). (F) Multicolor immunofluorescence staining of metastatic tumors for PD-1+ T-cell detection and quantification analysis of (G). * p< 0.05, ** p< 0.01. Scale bar=50μM. Error bars denoted s.e.m.

**Supplementary Table:**

**Table S1. Clinical features of the CRC-HM and CRC patients**

| **Characteristic** | **CRC-HM (%)**  **N=20** | **CRC (%)**  **N=20** | **p** |
| --- | --- | --- | --- |
| **Age (years)** |  |  |  |
| 40-60 | 10(50%) | 13(60%) | 0.522 |
| ≥60 | 10(50%) | 7(40%) |  |
| **Gender** |  |  |  |
| Male | 14(70%) | 15(75%) | 0.723 |
| Female | 6(30%) | 5(25%) |  |
| **Histology** |  |  |  |
| adenocarcinoma | 20(100%) | 20(100%) | - |
| **TNM stage** |  |  |  |
| I | 0 (0%) | 0(0%) |  |
| II | 0(0%) | 16(80%) | 0.000 |
| III | 0(0%) | 4(20%) |  |
| IV | 20(100%) | 0(0%) | 0.000 |
